# Electrokinetic propulsion for electronically integrated microscopic robots

**DOI:** 10.1073/pnas.2500526122

**Published:** 2025-07-15

**Authors:** Lucas C. Hanson, William H. Reinhardt, Scott Shrager, Tarunyaa Sivakumar, Marc Z. Miskin

**Affiliations:** ^a^Department of Physics and Astronomy, University of Pennsylvania, Philadelphia, PA 19104; ^b^Department of Electrical and Systems Engineering, University of Pennsylvania, Philadelphia, PA 19104

**Keywords:** microrobots, electrokinetic propulsion, micromotors

## Abstract

Semiconductor microelectronics are emerging as a powerful tool for building smart, autonomous sub-millimeter robots. Yet a number of existing microrobot platforms, despite significant advantages in speed, robustness, power consumption, or ease of fabrication, have no clear path toward electronics integration, limiting their potential for intelligence. Here, we show how to upgrade a class of self-propelled particles into electronically integrated microrobots, reaping the best of both platforms in a single design. Inspired by electrokinetic micromotors, these robots generate electric fields in a surrounding fluid, and by extension propulsive electrokinetic flows. The underlying physics is captured by a model in which robot speed is proportional to applied current, making design and control straightforward. As proof, we build basic robots at the 100-micron scale that use rudimentary, on-board photovoltaic circuits and a closed-loop optical control scheme to navigate waypoints and move in coordinated swarms at speeds of up to one body length per second. Broadly, the unification of micromotor propulsion with on-robot electronics invites future work to realize robust, fast, easy to manufacture, electronically programmable microrobots that remain operationally viable for months to years.

## Significance

Electrokinetic propulsion offers speed, simplicity, and reliable operation at the microscale, but, despite decades of research, current micromotors cannot incorporate on-board systems for sensing and information processing, limiting their usefulness. Here, we point a way forward by demonstrating electrokinetic microrobots whose propulsion is directly controlled by onboard electronics, namely photovoltaic cells. Although incorporation of complex circuits is reserved for future work, these initial demonstrations simplify design and control of electrokinetic microrobots, decoupling the chemical environment from the propulsive electric field, and operate in new environments like those with high conductivities or that lack specialized fuels. Long term, these actuators could enable fast, robust sub-millimeter robots that use onboard electronics to sense, think, and act all on their own.

Remarkable advances in electronics have set the stage for intelligent robots to shrink below the millimeter scale. In the past decade, circuit designers have pushed computing (1–3), memory (1, 2), and sensing (1, 4–7) systems into sub-millimeter dimensions, raising the prospect of microrobots that autonomously carry out intelligent decision making (8). Taking advantage of this progress, roboticists have demonstrated electronically integrated microrobots with increasingly impressive capabilities including reconfigurable gait patterns (9), two-way optical communication (10), and energy transfer with both light and radio frequency fields (9, 11–13). Miniaturization of both the circuits and actuators has dramatically reduced operating power, potentially enabling a 100 μm programmable robot capable of sensing and on-board computing to run on solar power harvested from ordinary daylight (9, 14, 15). These growing abilities suggest an emerging breed of microrobots able to sense and adapt to overcome uncertainty without the need for human supervision.

While prior work in electronically integrated microrobots has centered around a limited set of actuation schemes (12, 15), the enabling circuits have the potential to improve a number of microrobot designs. Electrical energy can be converted into a variety of different domains, suggesting that other approaches to movement, like those based on chemical (16), magnetic (17), or acoustic fields (18), could be brought within this framework by establishing on-robot electronic control over the underlying propulsion process. Building these bridges would enable individual parts for information processing and actuation to be mixed and matched to offset weaknesses and emphasize strengths, provided they meet basic electrical and manufacturing constraints.

Here, we take a step down this road, transforming electrokinetic micromotors into electronically controllable microrobot actuators. Already an inherently electrical process, electrokinetic propulsion offers unique advantages like high speed, low power, and easy fabrication (19, 20). Yet despite extensive work detailing the role of particle shape (21–25), solution chemistry (21, 25), ion type (21), and motor material (22, 25), no clear path has been established to integrate these motors with onboard systems for information processing. As a result, such motors are currently restricted to simple tasks with limited degrees of autonomy.

While typically the fields that drive motion are produced by specially tuned chemical reactions, here we show they can also be driven by on-robot semiconductor microelectronics, raising the possibility of integrating control, computation, and sensing on-robot. In this work, we keep the circuits rudimentary to facilitate characterization, demonstrating only power transfer and remote, optical control through silicon photovoltaics. However, even at this level, the fusion of electrokinetics with circuits brings reciprocal benefits. Compared to existing actuators for electronically integrated microrobots, electrokinetic propulsion offers faster speeds, longer lifetimes, and simplified fabrication. Conversely, compared to bare-bones self-propelled particles, electronics make the resulting robot more robust to chemical changes in its environment, easier to control, and clears a path for future work to integrate sophisticated systems for sensing and computation.

## 1. Results

**Fig. 1.**
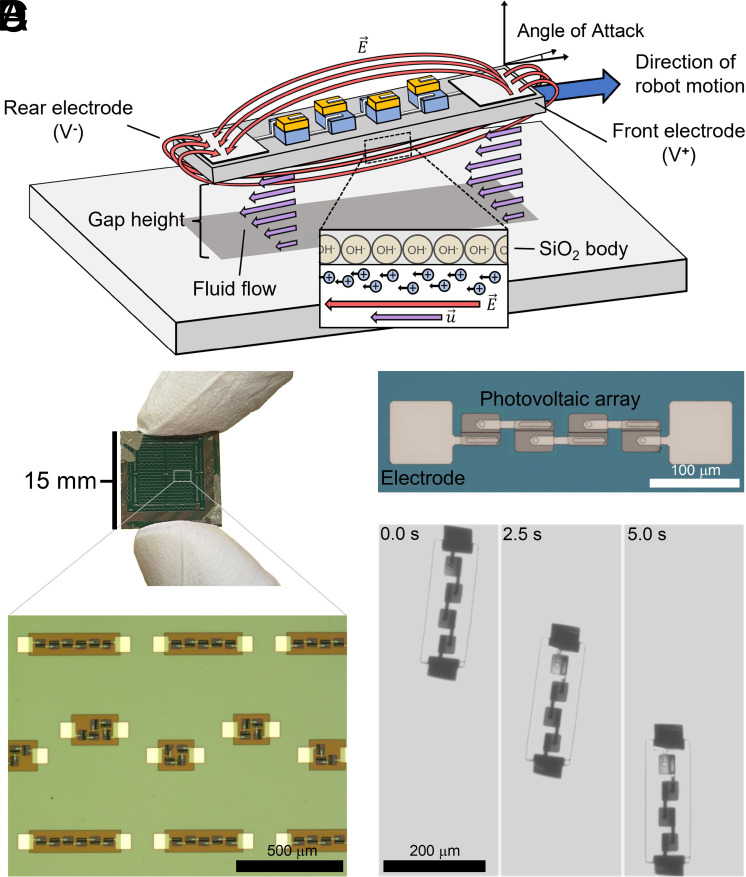
Electrokinetic propulsion for microrobots. (*A*) Schematic of the electrokinetic mechanism. Mobile ions in the electrical double layer migrate in the presence of an electric field, $\vec{E}$, and generate fluid flows around the device that cause locomotion. (*B*) Optical image of a silicon chip with hundreds of robots on it, with various shapes and PV numbers. Beneath it, a micrograph of various robot designs on chip, fabricated massively in parallel with 400 devices per 1.5 cm square chip (Scale bar, 500 μm.) (*C*) Micrograph of a 4 PV design with Ti/Pt electrodes at both ends of the device’s SiO_2_ body (Scale bar, 100 μm.) (*D*) Montage of a device moving under global microscope illumination in solution (Scale bar, 200 μm.)

The general mechanism behind electrokinetic propulsion is well established (26, 27) and depicted in Fig. 1*A*. The motor generates an electric field by feeding current into solution. This field pushes on nearby mobile charges (e.g., in the electrical double layer surrounding the motor and/or nearby surfaces or the diffusion layer). Movement of the charges is resisted by drag from the surrounding fluid, establishing a flow that moves the motor.

To realize an electronically controlled version, we build robots that generate propulsive electric fields using on-board photovoltaic cells (PVs) (Fig. 1*B*). We deliberately keep the circuit simple, stripping the robot’s electronics to the bare minimum to facilitate measurement and interpretation. The PVs, wired in series, feed current into the solution through 70 × 70 μm^2^ titanium-platinum electrodes at either end of the robot. Because the nominal voltage supplied by these cells is large enough to perform hydrolysis, the complete circuit is current limited by the incident light flux, allowing us to directly control the applied electric field with the illumination intensity (i.e., current and intensity are proportional to each other for a photovoltaic operating near the short-circuit current). For insulation, the PVs and associated wiring are covered in a layer of photoresist. All of these parts are fabricated massively in parallel (*SI Appendix*, section A) with a previously developed, fully lithographic protocol (9, 11), enabling several hundred devices per 1 cm chip as seen in Fig. 1*B*.

**Fig. 2.**
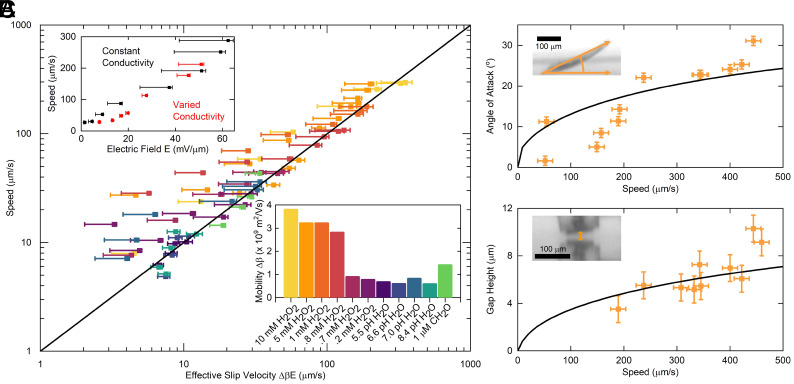
Characterization of propulsion mechanism. (*A*) Speed vs. electric field behavior for a variety of chemical environments scaled by the robot’s effective mobility Δβ (that is V=ΔβE; see *Materials and Methods*). The black line is unity. The *Upper Inset* depicts speed vs. electric field data for a robot in 5 mM hydrogen peroxide, where conductivity and applied current are independently varied. The *Lower Inset* details the variation of the effective mobility for different solution compositions. (*B* and *C*) Data for the angle of attack and gap height vs. speed, respectively. The black curves are the results of numerical fitting of the data to our fluid model (*SI Appendix*, section C). The *Insets* show micrographs of a moving robot viewed from the side and front used to produce the data (Scale bars, 100 μm, *Materials and Methods*.) Note that the same robot and chemical environment (5 mM peroxide) were used to measure all data in panels (*B* and *C*).

Once released into solution and illuminated, robots move at a steady speed, oriented with the positive electrode in the front (Fig. 1*C* and Movie S1). Negatively buoyant, the robots move along the bottom surface of their container. We independently measure the current produced by the PVs under various illumination conditions (*Materials and Methods*) and find the robot’s speed is proportional to current density and inversely proportional to solution conductivity, *σ* (*SI Appendix*, Fig. S1). Indeed, when speed is plotted against the electric field (i.e., the ratio of current density to conductivity), the data collapse to a line, as shown in the *Upper Inset* of Fig. 2*A*.

Aside from conductivity, we find propulsion speed is largely independent of the surrounding chemical environment. Fig. 2*A* shows robots can move in a variety of solutions, including two decades of different hydrogen peroxide concentrations, pure deionized water (σ= 250 nS/cm), salt solutions (σ=10 μS/cm), pH buffers (5.5 to 8), and formaldehyde. We also find robots move on a variety of substrates, including polystyrene, oxygen plasma cleaned glass, positive charge functionalized glass, SU-8 photoresist films, platinum, and through microfluidic channels (Movie S2). The only chemical condition that we found that reliably turns off propulsion is high solution conductivity (≫10 μS/cm), which places propulsive fields into an inaccessible regime for the robot’s power budget.

When moving, the relationship between speed and electric field is consistently linear, with the slope alone coupled to changes in the chemical environment. In Fig. 2*A*, we force the speed and field data for each solution to collapse to a unity-slope line with the necessary scaling factors, i.e. the effective mobilities, detailed in the *Inset* graph. In general, the effect of the chemical environment is small: The largest ratio of speed to field strength (10 mM hydrogen peroxide) and the lowest (deionized water) only differ by a factor of roughly 4. By contrast, the speed of the robots can be changed by more than two orders of magnitude through electronic control of the current, effectively decoupling chemical conditions from the robot’s ability to move.

To better analyze the underlying fluid dynamics, we image propulsion from the side, directly measuring the robot’s three degrees of freedom: speed, angle of attack, and gap height above the substrate (Movie S3). The robot’s angle and gap are too large to be generated by buoyancy effects nor do we see bubble formation, suggesting the dominant forces are hydrodynamic in nature (see *SI Appendix* for further discussion). As shown in Fig. 2, each parameter increases as the field gets larger. Given the three kinematic constraints from force and torque equilibrium, a model can be constrained to predict a unique solution for the robot’s configuration.

We find three physical ingredients are sufficient to predict the trends in Fig. 2. First, we make a lubrication approximation, assuming fluid forces are largest in the small gap between the robot and substrate. All other forces from the fluid are lumped into small phenomenological terms derived by symmetry considerations. Second, we assume the electric field dominantly points along the axis running from the positive to negative electrode. Third, we use the “standard model” of electrokinetics (26, 27), which couples the fluid to the electric field by imposing a boundary slip condition $\Delta U=\beta \vec{E}$, where *β* is the slip coefficient for that surface. We stress this model is distinct from those currently used to describe micromotors because we are operating at a much larger length scale and at much larger field strengths: Whereas micromotors can have features on the order of the Debye length (here ∼10 to 100 nm), our robots are over three orders of magnitude larger in size and thus in the “thin Debye layer” limit. Likewise, *β* is the only parameter in this model that depends on the chemical environment, unlike existing models where chemical reactions, flows, and fields are strongly coupled (28, 29). In other words, the model is largely agnostic to the specific ion type involved, reflecting the fact that the electric field is regulated by an on-robot galvanostat.

We numerically solve the governing fluid and electrical field equations in the gap under the robot and impose force and torque balance to identify equilibrium angle and gap height at a given electric field strength. Fitting the model produces the black traces in Fig. 2 *B* and *C*, which agree with the data (30). Note both datasets, with 21 total data points, are fit simultaneously to the same three model parameters, namely the phenomenological shear and torque parameters and the difference in mobility between the robot body and the substrate (Δβ). Further, the fit parameters are consistent with the underlying physics. For instance, the difference in mobility between the robot’s body and the substrate, when fit, is within the range of literature values for the mobility of silicon dioxide and polystyrene in aqueous solutions at similar pH values (31, 32). The sign of the fit values, which sets the propulsion orientation, suggests the dissociated charges near the glass surface of the robot’s body are predominately responsible for propulsion (as depicted in Fig. 1*A*), consistent with the observation that robots move the same way on a variety of substrates.

Compared to existing micromotors, those powered with electronics see several improvements. Many motors such as bimetallic nanorods or Janus particles, while elegant in their simplicity, operate at small sizes, low energy scales (33–35), and are strongly tied to environmental chemistry for propulsion. By contrast, electronic control of the current flowing through solution lifts these constraints, enabling operation in a range of environments and at larger sizes and energy scales. As noted earlier, the robots presented here operate at fields nearly two orders of magnitude larger than chemically driven motors (22, 34, 35). By extension, the robot retains the ability to propel at roughly one body length per second, even though it is more than one hundred-fold larger in size. The increase in size is noteworthy, since by operating at 100 microns instead of a mere 1 micron, such devices have a realistic path to integrating systems for sensing or computation. Similarly, electronically controlled designs can operate reliably even in the absence of chemical fuels like hydrogen peroxide and/or in solutions over one hundred-fold higher in conductivity because the robots actively set the current passing through solution to meet the field strength needed to drive propulsion. Finally, we note that although the power efficiency of these motors is low (∼10^−8^), the nominal power value is small enough (100 nW) to be integrated with a variety of power supplies beyond photovoltaic energy conversion (36).

**Fig. 3.**
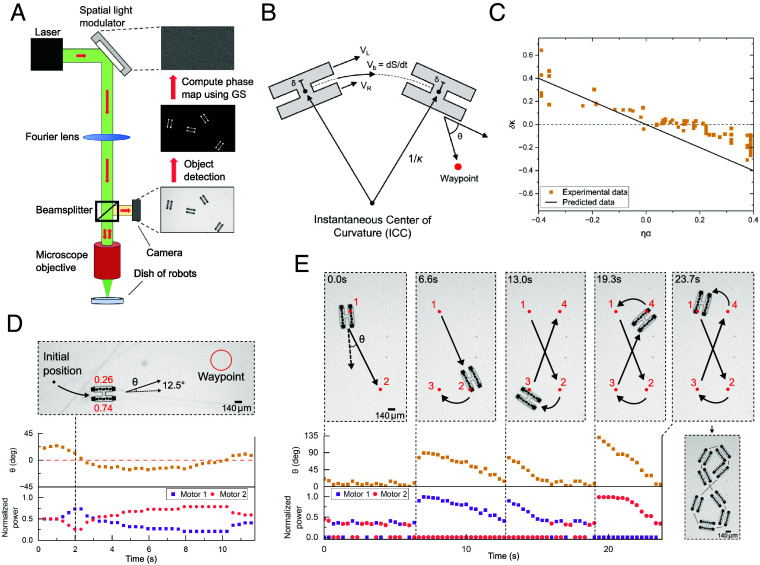
Control and kinematics of a two motor robot. (*A*) A closed-loop optical system for automated control over robots. After each frame capture, a computer performs object detection and generates a new optical pattern to control robots. (*B*) A diagram depicting the variables involved in our control laws. (*C*) As expected for differential drive kinematics, the curvature *κ* nondimensionalized using the robot width *δ* is proportional to the normalized difference between motor velocities *η* when accounting for optical effects *α* (see *SI Appendix* for discussion on stray light correction, *SI Appendix*, section D). The black line has slope = −1. (*D*) Implementation of a differential drive controller, where power to each motor is simultaneously adjusted in order to place the robot at specific locations. See Movie S4. (*E*) Path trace of a controller that adjusts only the most misaligned motor to pilot the robot around a lemniscate. See Movie S5.

These motor performance advantages are further compounded by the fact that even simple electronics can facilitate various control schemes. To demonstrate this potential, we show independent steering of multiple microrobots, using an external light source to regulate motion. We go from the linear motion in Fig. 1*A* to turning by joining two motors together and independently varying the current supplied to each. For the simple test circuits built from PVs, we modulate current directly using a spatial light modulator (SLM) and a closed-loop control scheme, as shown in Fig. 3*A*. The SLM generates optical patterns of high and low intensity light to selectively power individual motors (*Materials and Methods*) and a computer generates new optical patterns based on the image data and user prescribed control laws. This system, though rudimentary, runs autonomously, tracking robots and shooting light at them as they move.

By studying the robot’s behavior under different proportions of power, we find the two joined motors obey differential drive kinematics. Specifically, the left and right side of a robot generate flows with different velocities ($V_{L}$ and $V_{R}$, respectively), resulting in a forward body velocity equal to their average and a curvature proportional to their normalized difference $\eta =\left(V_{L}-V_{R}\right)/\left(V_{L}+V_{R}\right)$ (Fig. 3*B*) (37). Indeed, a comparison of the predictions for differential drive kinematics to the measured data is shown in Fig. 3*C*. Accounting for imperfect focusing of the light pattern (*SI Appendix*, section D), we find agreement without fit parameters.

Differential drive systems offer a variety of control laws that reliably position robots in space and time. As an example, we implement two (38, 39) (*SI Appendix*, section E) to direct our robots through a series of waypoints. Both controllers proportionally adjust motor power using the angle between the robot’s heading and a vector that points from the robot’s center of mass to the target position, denoted by *θ* in Fig. 3. One controller adjusts both motors simultaneously to direct the robot to a target location (Fig. 3*D* and Movie S4) while the other adjusts the power on the motor farther from alignment to steer the robot through a lemniscate pattern (Fig. 3*E* and Movie S5).

**Fig. 4.**
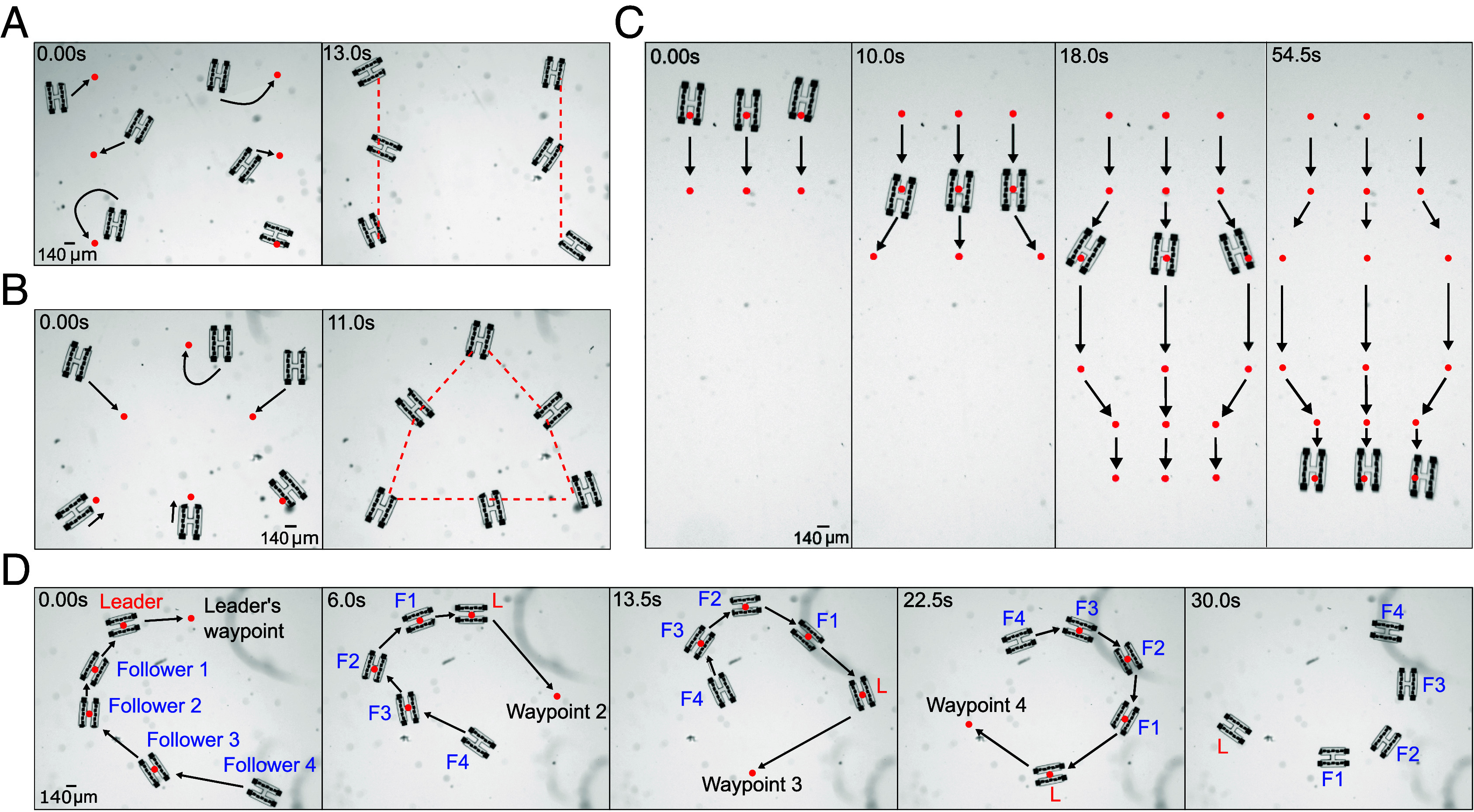
Addressability and swarming behavior enabled by circuits. (*A* and *B*) The control program can be given a list of target positions in order to guide each robot to its nearest waypoint. (*C*) Robots can be assigned individual lists of waypoints in order to trace out separate paths in unison. (*D*) Waypoints can also be dynamic and change at each timestep. Shown in blue, “follower” robots are assigned the location of another robot in the system as a waypoint. This rule results in chain-like structures following the “leader,” shown in red.

Thanks to the use of on on-board circuits, parallel control of many microrobots becomes straightforward. This stands in contrast to many existing micromotors, which can be harder to localize and control when reliant on energy distributed across the environment for actuation (40, 41). To demonstrate, in Fig. 4 *A* and *B*, we generate a list of waypoints and digitally assign each one to the nearest robot. On command, the robots rearrange independently to form the user-defined shapes, here lines and triangles (Movies S6 and S7). Furthermore, each robot can be given its own list of waypoints in order to have multiple robots perform sequences of separate tasks. Fig. 4*C* and Movie S8 show three robots swimming in unison on different paths. Last, paths can be dynamic, evolving with the swarm. In Fig. 4*D*, we create a chain of robots by assigning each robot to follow a neighboring device (Movie S9).

We note this approach could be scaled up by simply improving the SLM (*SI Appendix*, section F). Alternatively, the underlying kinematics and control laws can be used regardless of the mechanism that modulates propulsion, suggesting the SLM and tracking system could be replaced by on-robot circuits for control and sensing. As an added benefit, replacing the optical system would eliminate imperfections in the current strategy from stray light, resulting in kinematics that more closely match the ideal behavior.

## 2. Discussion

Akin to self-propelled particles, the simplicity of electronically controlled electrokinetic actuators confers speed and robustness. We find robots can last over a year in solution, can be transferred with macroscopic pipettes upward of one hundred times, and can be dried and reimmersed without loss of function. This stability marks a major improvement over other robots we have built using electrochemical actuators (9, 11) or bubble generators, which are far easier to damage and typically last for a few weeks at most. Further, electrokinetic actuation is appreciably easier to implement, requiring only a single step of lithographic patterning to be added onto circuits. The actuator itself is a chemically stable, but more or less arbitrary metal electrode, requiring few material considerations, no atomically thin material layers, and no 3D self-folding construction (9, 42, 43). These improvements in stability, fabrication, and performance are important as microrobots move toward real-world applications.

Crucially these advantages can be realized without sacrificing the capacity to integrate electronics. Further work could help extend this best-of-both-worlds scenario, in which the high speeds and robust performance inherent to micromotors is mixed with the potential for sensing and computational circuits. One route would be to seek out other self-propelled particle modalities that can be brought under electronic control. For instance, alternating current electrokinetic techniques are a natural extension of the direct current mechanism used here.

Alternatively, future work can readily integrate more sophisticated systems for sensing and computation. To do so, electronics must simply regulate the current between pairs of electrodes. Indeed, using the designs and physics explored here as a foundation, we explicitly demonstrate on-robot intelligence in a companion publication (44), integrating sensing, communication, memory, computation, and closed-loop feedback on a single sub-millimeter electrokinetic robot. Combined, these developments point to an exciting, near-term future, in which microrobots can use a wide variety of actuation mechanisms to carry out long-term, complex tasks without human supervision.

## 3. Materials and Methods

### 3.1. Microrobot Fabrication.

Microrobots are fabricated using standard semiconductor processing techniques on p-type silicon on oxide (SOI) wafers, consisting of a 2 micron thick device layer, a 500 nm silicon dioxide layer, and 500 microns of handle silicon. Photovoltaics are fabricated by diffusing n-type dopants into the top layer of silicon, and then plasma etching mesa structures to allow the formation of contacts to both the n-type and p-type layer. Conformal silicon oxide is deposited to insulate photovoltaics, and contacts are formed via HF etching and sputtered titanium and platinum. Subsequently, interconnects between the photovoltaics, as well as electrodes, are formed from sputtered titanium and platinum. The photovoltaics and wires are insulated with SU8, and the bodies of the robots are defined by plasma etching the silicon dioxide layer. The robots are released from the wafer by sputtering an aluminum support film on top of the robots, and then etching the handle silicon from underneath using XeF_2_ vapor. The support film is then etched in aluminum etchant A, resulting in released individual robots. Our fabrication process is illustrated in *SI Appendix*, Fig. S3, and discussed in detail in *SI Appendix*, section A.

### 3.2. Characterizing PVs.

PVs were characterized by probing with tungsten microprobes (Signatone SM-35) and performing current-voltage sweeps at various illumination intensities using a Keithley source meter (2450). Individual PVs output photocurrents on the order of 100 nA and open circuit voltages of approximately 500 mV when illuminated with a white light source (Thorlabs Solis 1-D) at maximum power in an upright microscope (Olympus) (*SI Appendix*, Fig. S4). Open circuit voltages sum for multiple PVs wired in series, enabling the devices to operate effectively as current sources in solution (*SI Appendix*, Fig. S5).

### 3.3. Solution and Substrate Preparation.

Hydrogen peroxide solutions were prepared by dilution of 30% hydrogen peroxide (Fisher H325-500) with DI water from our facility with an initial conductivity of 250 nS/cm. At appreciably higher concentrations, we found bubble formation occurs, disrupting propulsion. Conductivity of solutions was adjusted with addition of sodium nitrate (Sigma-Aldrich S5506-250G) and sodium nitrite (Fisher S347-500) at various concentrations. Buffer solutions were prepared by dilution of various buffers (Fisher SB107-500, Fisher SB115-500, Fisher SB101-500), and 50% sodium hydroxide (Transene 1310-73-2). Formaldehyde solutions were prepared by dilution of 37% formaldehyde stabilized with methanol (Fisher F79-500). Solution pH was measured with a Hach Pocket Pro pH meter (PN 9531000) and solution conductivity was measured with a Hannah Instruments pure water conductivity meter (HI98197). Polystyrene substrates used were sterile 60 × 15 mm petri dishes (VWR International 25384-D92). Glass substrates used were microscope slides (Fisher 12-549-3 and Thorlabs MS10PC1, for negative and positive surface functionalization, respectively). Platinum substrates used were made by sputtering 20 nm of titanium and 40 nm of platinum on a 25 mm glass coverslip (Deckglaser 100). SU-8 substrates and microfluidic channels used were made by spinning SU-8 2050 photoresist on a 4 inch borofloat wafer at 2,000 rpm for 40 s, soft baking 9 min at 95 ^°^C, exposing regions around channels with a mask aligner through an I-line filter, post baking for 7 min at 95 ^°^C, developing in SU-8 developer for 7 min with agitation, rinsing with IPA, and hard baking for 5 min at 200 ^°^C.

In all experiments, the most significant factor that impedes propulsion was an uncontrolled increase in solution conductivity, usually via the introduction of ionic contaminants to the solution. Specifically, when the conductivity is raised too high, the robot cannot provide sufficient current to maintain its motion with the onboard photovoltaic cells (i.e. the required fields for movement are outside the IV operating range for the underlying circuitry). To mitigate this effect, all surfaces that met the solution were rinsed in DI water multiple times to dissolve and remove contaminants prior to experiments.

### 3.4. Measuring the Speed vs. Current Response of a Single Motor Robot.

To measure the current driven through solution by a robot under various illumination intensities we fabricate two test chips: a test chip with PV circuits identical to those on the robots and a test chip with identical electrodes. The electrode test chip is immersed in the desired solution under a stereoscope (ZEISS SteREO Discovery.V8), and the PV test chip is placed under an upright optical microscope in air (Olympus). We probe the circuits on the PV chip with tungsten probes (Signatone SM-35) under illumination in reflection mode with a variable intensity white LED source (Thorlabs Solis-1D). We probe the electrodes on the electrode chip with insulated Pt/Ir probes (Microprobes for Life Science PI20031.5A5). We then wire the probes from each chip in series, such that the PV circuit is driving current through the electrodes in solution, which we measure at various light intensities with a low noise current preamplifier (Stanford Research Systems SR570). The results of these measurements are detailed in *SI Appendix*, Fig. S6. We then place a single motor robot in the same solution used to measure the current, and illuminate the robot at the same light intensities in order to measure the speed. We take 10 frames per second image sequences of the robot in motion and compute the center of mass of the robot in each frame through image thresholding and particle detection (ImageJ). The position data is smoothed by convolution with a Gaussian kernel, and the instantaneous speed is calculated for each frame as the magnitude of the position change divided by the time between frames. The reported speed is the average of all instantaneous speeds for the duration of the experiment, usually a few seconds. To estimate the mobility coefficient for each solution, the speed vs. field graphs are fit via linear regression, with the speed at zero field constrained to be zero. The data in Fig. 2*A* are scaled by the effective mobility to force data collapse.

### 3.5. Measuring Angle of Attack and Gap Height.

To measure angle of attack and gap height, a two motor robot is placed on a piece of polystyrene in a glass cuvette (Thorlabs CV10Q35A), and imaged simultaneously from the side using an adjustable magnification microscope (Olympus) in transmission mode, and from above using a stereoscope (ZEISS SteREO Discovery.V8), while illuminated at various intensities with a ring light (Schott S80-55). The speed of the device is measured using image sequences from the stereoscope, and the angle of attack and gap height are measured using image sequences from the side mounted microscope.

### 3.6. Estimate of Buoyant Forces.

Assuming a robot weight on the order of 1 nN, a bubble containing gas at standard temperature and pressure would need to be roughly 100 microns in radius to exert sufficient force to overcome gravity and sustain the observed gap between the robot and substrate. Bubbles above the resolution limit of our imaging apparatus (order R<1 μm) are not observed, implying that bubbles are unlikely to play a major role in propulsion.

### 3.7. Forming Computer Generated Holograms.

To form optical patterns in the microscope field of view, we mimic setups that are capable of reconstructing 2D or 3D holograms by displaying grayscale phase maps on an SLM (45–47). Using a Holoeye LETO-3 SLM in a phase-modulation scheme, we run the Gerchberg–Saxton (GS) algorithm on a grayscale image containing the optical pattern we want to create (48). This algorithm extracts a phase map that will diffractively reconstruct the target optical pattern in the far-field plane of the microscope after passing through a lens (49, 50). Additionally, to handle swapping between the coordinate system of the microscope FOV (5,320 × 3,032 pixels) and the SLM display (1,920 × 1,080 pixels), we generate an affine transformation matrix using an automated calibration program. This program places noncollinear points with known (x,y) coordinates on the SLM, detects the corresponding $\left(x^{\prime },y^{\prime }\right)$ points in the microscope FOV, and generates a transformation matrix to map $\left(x,y\right)↔\left(x^{\prime },y^{\prime }\right)$. At each timestep, we apply this matrix to the output of the GS algorithm to generate a phase map for the SLM that recreates the desired optical pattern in the microscope FOV with the correct scale, rotation, and shear. Further, as shown in Fig. 3*A*, the SLM is tilted at an angle relative to the incident laser light. This skew steers the 0th order spot, which cannot be modulated by the SLM, off the center of the optical axis. We then apply a blazed grating to the phase map extracted by the GS algorithm to bring the first order diffraction pattern back to the center of the optical axis and into the backplane of the microscope (51). While there is error in this system associated with contour detection, calibration, and imperfect focusing of light, Figs. 3 and 4 demonstrate that these effects do not significantly alter the kinematics. As such, we estimate our accuracy to be within 40 um, the approximate side length of a photovoltaic.

### 3.8. Object Detection.

Robot detection is performed in the closed-loop cycle pictured in Fig. 4*A*. Images from a USB camera (Basler Ace2 USB Camera) are sent to a Python script where robot positions and engine locations are determined by using adaptive thresholding in OpenCV (52) to extract contours. Tracking of individual robots frame-to-frame is done using Norfair (53). We find this method works at a range of microscope exposure parameters and is capable of tracking entire robots, individual engines, or even PVs depending on the thresholding values used. Although there is variability in the exact size and shape of contours that result from the thresholding, Figs. 3 and 4 demonstrate empirically that this is not prohibitive to robot tracking or controllable motion.

## Supplementary Material

Appendix 01 (PDF)

Dataset S01 (XLS)

Dataset S02 (XLS)

Dataset S03 (XLS)

Dataset S04 (XLSX)

Dataset S05 (XLSX)

Dataset S06 (XLSX)

Dataset S07 (XLSX)

Movie S1. A single motor under microscope illumination. (This video is in real time).

Movie S2. A single motor robot travels through an SU8 channel under microscope illumination. (This video is in real time).

Movie S3. A side-on view of a two motor robot under illumination. Due to asymmetry in the motors, the robot drives in a circle. At the end of the video, the driving illumination is turned off and the robot stops moving. (This video is in real time).

Movie S4. A controller autonomously pilots a robot to a waypoint by trimming the power on each engine. (This video has been sped up by 2×).

Movie S5. A controller autonomously pilots a robot through a series of waypoints to trace out a lemniscate. (This video has been sped up by 2×).

Movie S6. Given a list of target locations, robots autonomously travel to the nearest waypoint to form user-defined shapes. Shown here is a rectangle. (This video has been sped up by 2×).

Movie S7. Given a different list of target locations, robots rearrange to form other user-defined shapes such as triangles. (This video has been sped up by 2×).

Movie S8. Robots are assigned an individual list of waypoints to trace out separate paths simultaneously. (This video has been sped up by 2×).

Movie S9. Robots are assigned dynamic waypoints such as the location of another robot in the system and form a chain-like structure in which they follow their nearest neighbor. Additionally, the controller prevents robots from getting too close together, resulting in the stop-and-go behavior seen in this video. (This video has been sped up by 2×).

## Data Availability

Some study data are available: All data included in the main text and supplemental graphs are included as *SI Appendix* datasets. Image data are included as supplemental movies due to size constraints, but raw image stacks can be made available upon request. All of the model code to solve for pressure, integrate the ODE, and fit the data is hosted on Zenodo at https://doi.org/10.5281/zenodo.15742030 (30).
