# Supplementary material for: Electrokinetic propulsion for electronically integrated microscopic robots
Source: Proc Natl Acad Sci U S A. 2025 Jul 15;122(29):e2500526122. doi: 10.1073/pnas.2500526122 (PMC12305017; doi:10.1073/pnas.2500526122)
Supplement: Supplementary file 1 — Appendix 01 (PDF) [file pnas.2500526122.sapp.pdf]

PNAS

Supporting Information for

Optically controllable electrokinetic microrobots

Lucas C. Hanson, William H. Reinhardt, Scott Shrager, Tarunyaa Sivakumar, Marc Z. Miskin

Marc Z. Miskin
E-mail: mmiskin@seas.upenn.edu

This PDF file includes:

Supporting text
Figs. S1 to S7
Legends for Movies S1 to S9
SI References

Other supporting materials for this manuscript include the following:

Movies S1 to S9

Supporting Information Text

A. Full Fabrication Protocol. For our fabrication process (see Supplemental Fig. S3), we use silicon-on-insulator wafers from Ultrasil corporation with a 2 μm device layer (p-type doped, 0.1 Ohm-cm), 500 nm silicon dioxide layer, and 500 μm silicon handle thickness. 15 mm chips are diced from a 4 inch wafer using a dicing saw, spin coated with P509 spin-on glass from Filmtronics, and postbaked for 20 minutes at 120°C. We use a rapid thermal annealer to diffuse the P509 (n-type phosphorus) dopants by annealing for 30 minutes at 800°C in an N_2 environment to form a layer of n-type silicon above the p-type silicon. Once the doping is complete, we remove the spin-on glass by immersing the chip in 6:1 buffered oxide etch (BOE).

We build the photovoltaics (PVs) through a series of etching and metallization steps. Before starting, we measure the thickness of the silicon device layer precisely at four different locations across the sample using a Filmetrics reflectometer. We spin on Shipley 1813 photoresist and expose using a mask aligner. Next, we develop the exposed photoresist in AZ300 MIF developer for 50 seconds and O_2 plasma clean the sample at 150 W for 90 seconds. We etch in an RF plasma SF_6/O_2 environment for 40 seconds to expose the p-type device layer silicon and then measure the thickness of the silicon again to confirm an etch depth of at least 1 μm. We strip the photoresist by ultrasonicating in heated PG remover for 10 minutes and O_2 plasma cleaning at 150 W for 90 seconds.

Our second etch step forms discrete PVs. First, we spin on an HMDS/AZ3330 photoresist stack and expose using a mask aligner. Next, we develop the exposed photoresist in AZ300 MIF developer for 50 seconds and O_2 plasma clean at 150 W for 90 seconds. We adhere our samples to a carrier wafer using CrystalBond and perform an anisotropic Bosch etch with a deep reactive ion etcher. Using the insulating oxide layer of the SOI as an etch stop, we completely clear away the device layer silicon in the exposed regions to form discrete PV cells. We remove the samples from the carrier wafer by ultrasonicating in heated (70°F) water and then strip the photoresist by ultrasonicating in heated PG remover for 10 minutes. We then O_2 plasma clean the samples at 150 W for 90 seconds.

Next, we make metal contacts on the p-type and n-type silicon. We start by insulating the PVs by depositing a 20 nm conformal layer of silicon dioxide using an atomic layer deposition (ALD) tool. We spin on HMDS/AZ3330 photoresist, expose in a mask aligner, and develop in AZ300 MIF developer for 50 seconds to form small openings in the photoresist to the p-type and n-type silicon. We O_2 plasma clean at 150 W for 90 seconds then immerse the samples in 6:1 BOE for 15 seconds to remove the insulating oxide layer in the exposed regions. Before the native oxide layer regrows on the exposed silicon, we sputter 20 nm of titanium and 40 nm of platinum over the entire sample. We then perform a lift-off by stripping the AZ3330 photoresist in heated PG remover with ultrasonication, followed by an O_2 plasma clean at 150 W for 90 seconds. Finally, we anneal the contacts in a rapid thermal annealer for 5 minutes at 400°C in an N_2 environment.

To wire the PVs together and form the device's electrodes, we spin on HMDS/AZ3330 photoresist, expose in a mask aligner, develop in AZ300 MIF developer for 50 seconds, and O_2 plasma clean at 150 W for 90 seconds. We sputter 20 nm of titanium followed by 40 nm of platinum and perform a liftoff process in heated PG remover with ultrasonication to form interconnects between the PVs and electrodes at either end of the PV line.

Next, we define the shape of the robot's body by etching the silicon dioxide layer of the SOI in the regions between robots. We spin on HMDS/AZ3330, expose in a mask aligner, and develop in AZ300 MIF for 50 seconds. We O_2 plasma clean at 150W for 90 seconds and perform a 25 minute CF_4 etch to remove the exposed silicon dioxide. We then verify that the oxide has been removed using a Filmetrics reflectometer and strip the photoresist in heated PG remover with ultrasonication.

In order to insulate our electronics stack from the surrounding solution, we spin coat SU-8 2005 epoxy at 3000 RPM for 30 seconds with a 1000 RPM/s ramp rate. We soft bake the samples at 65°C for 1 minute and then at 90°C for 2 minutes before stripping the edge bead and exposing in a mask aligner through an I-line filter. After the sample is exposed, we bake the chips at 65°C for 1 minute, transfer to a separate hotplate to bake at 95°C for 3 minutes, develop for 60 seconds in SU-8 developer with agitation, followed by a rinse in IPA. We then hard bake the SU-8 at 65°C for 1 minute, transferring the samples to a 95°C hot plate, and ramping up to 150°C where the samples are held for 5 minutes. Subsequently, we turn off the hot plate and allow the sample to cool to room temperature to relieve thermal stress in the epoxy. We O_2 plasma clean at 150 W for 90 seconds.

We then sputter 200 nm of aluminum, followed by 10 nm of alumina deposited via ALD. We spin on an HMDS/AZ3330 stack, expose in a mask aligner, and develop in AZ300 MIF for 120 seconds with constant agitation to form small openings to the alumina/aluminum around the perimeter of the robots. After an O_2 plasma clean at 150 W for 90 seconds, we post-bake the samples at 115°C for 2 minutes and place them in aluminum etchant A for at least 20 minutes to clear the alumina/aluminum stack and expose the handle silicon. We remove the photoresist and run a XeF_2 etch to remove the underlying silicon through the holes patterned in the aluminum and suspend the devices in air. Lastly, we etch away the alumina/aluminum stack holding the devices by placing the samples in 10:1 diluted aluminum etchant A overnight. Once the aluminum is removed, we pipette the free floating devices out of the etchant and into deionized water for storage.

B. Estimating field strength and mobility coefficient. The magnitude of the field in the solution at the electrode interfaces can be computed simply as $J/\sigma = I/a^2\sigma$, where I is the current generated by the PVs, a is the side length of each electrode, and σ is the solution conductivity. However, since the pressures and shears that drive propulsion occur in the fluid gap underneath the robot, we seek to estimate the field strength due to current flowing in this region. This can be achieved by first estimating the voltage drop in the solution between the electrodes. For the simple case of a 2D electrode on an insulating substrate with characteristic size a and current density J, the potential at the electrode interface relative to a point at infinity can be found from the Laplace equation to be $\Delta\phi \sim I/a\sigma$. By the linearity of solutions to the Laplace equation, this result holds for the

case where a grounding electrode of the same size lies at some lateral distance l from the first electrode, yielding an expression for the potential drop between them. We can thus approximate the field magnitude as $E \sim \Delta\phi/l = I/a\sigma l = Ja/\sigma l$. In other words, the propulsive field strength is equal to the field magnitude at the electrode interface reduced by a factor a/l. It is this propulsive field by which the value of the electrokinetic mobility is determined.

C. Modeling microrobot propulsion. From the side imaging of robots, we observe that the fluid gap underneath the robot is significantly smaller than the robot's length, l or width, w. This allows for a considerable simplification of both the electrical and fluid parts of the problem by focusing on the field and flows within the gap, as these should dominate the overall behavior of the system.

We approximate the gap profile as a linear function along the direction of transit (here the x-axis) and as a constant along the orthogonal axis (the y-axis, defining the robot's width). The z-axis points along the gap direction. A schematic of our coordinate system is included as SI Figure S7.

In the electrical domain, we assume electrical current flowing across the robot's body is governed by Ohm's law $\nabla^2\phi = 0$. Since there is a no flux boundary for current both at the lower substrate and the robot's body, we expect current flow along the z-axis (i.e. along the gap direction) to be negligible. Thus, we integrate the potential from the top to the bottom of the gap, reducing the electric field equation to

$$\partial_x(h(x)\partial_x\phi) + \partial_y(h(x)\partial_y\phi) = 0 \qquad [1]$$

Ignoring field variations across the robot's width (y-axis), as no bias is applied along this direction, we find the field is approximately governed by

$$\partial_x(h(x)\partial_x\phi) = 0 \qquad [2]$$

Enforcing that the potential equals the applied electrode potential at either end of the robot leads to the solution

$$\phi(x) = (\Delta\phi)\left[\frac{\log[h(x)]}{\log[h_+/h_-]} - \frac{(h_+ + h_-)}{2\alpha}\log\left[\frac{h_+ + h_-}{2}\right]\right] \qquad [3]$$

where h_+ is the largest height of the gap, h_- is the lowest and $(\Delta\phi)$ is the applied potential drop. We note the first term conveys all the relevant physics since it is the only part of the expression with spatial dependence and the field alone contributes to propulsion. The second term is simply a reference potential chosen so that in the limit $\alpha \to 0$ the potential is approximately $\phi(x) \approx (\Delta\phi)x/l$.

Turning to the fluid domain, forces on the robot are computed by contracting the fluid stress tensor, $\sigma_{ij} = -p(x)\delta_{ij} + \mu(\partial_i u_j + \partial_j u_i)$, with the normal vector on the robot's body, $\hat{n} = \alpha/\sqrt{1+\alpha^2}\hat{x} - 1/\sqrt{1+\alpha^2}\hat{z}$. Here p is the pressure, u_i is the velocity of the fluid flow in the ith direction, and μ is the fluid viscosity. In the lubrication approximation, we assume that p is independent of z, that the dominate viscous stress term is $\partial_z u_x$, and that the fluid velocity along the x direction is given by $u_x = \frac{1}{2\mu}\partial_x p(z^2 - zh(x)) + (V + \Delta\beta E_x)z/h + \beta_- E_x$, where $\Delta\beta$ is the difference in surface slip coefficients, β_- is the slip coefficient of the surface under the robot, and $E_x = -\partial_x\phi(x)$. The resulting lift, drag and torque equations respectively read (1–3)

$$f_z = \langle p\rangle + \mu\alpha\langle(V + \Delta\beta E_x)/h(x)\rangle \qquad [4]$$

$$f_x = \frac{\alpha}{2}\langle p\rangle + \mu\langle(V + \Delta\beta E_x)/h(x)\rangle \qquad [5]$$

$$\tau = \langle xp\rangle + \mu\alpha\langle(V + \Delta\beta E_x)x/h(x)\rangle \qquad [6]$$

where angle brackets denote integration over the robot's area $\langle p\rangle = \int_{-l/2}^{l/2} dx \int_{-w/2}^{w/2} dyp$. The right hand sides represent forces from the lubrication zone, whereas the left hand side represents any external forcing.

Solving the fluid forces requires computing the pressure under the robot. Within the lubrication approximation, the pressure satisfies the Reynolds equation (1–3):

$$\frac{1}{12\mu}\partial_i(h^3\partial_i p) = \frac{1}{2}\partial_i(h(u_i^+ + u_i^-)) + u_z^+ - u_z^- - u_i^+\partial_i h \qquad [7]$$

where u_i^+ represents a fluid velocity in the ith direction on the upper surface, u_i^- denotes fluid velocities on the lower surface and contracted indices sum only on the x and y directions (i.e. the pressure is a function only of x and y).

To simplify the calculation, we exploit the linearity of low Reynolds number flow and split the pressure into two contributions: one from the translations and rotations of the robot's body (p_m) and the other from the electric field induced boundary slips (p_{ek}). These different boundary conditions for the fluid each set different terms on the right hand side of equation 7.

For the flow from rotation and translation, the boundary conditions simplify considerably. Here all lower surface velocities $u_i^- = 0$ while on the upper surface, there is motion from rigid translation in the x and z directions and a spatially varying velocity due to rotation around the center of mass. Specifically, $u_x^+ = V$ and $u_z^+ = U + x\dot{\alpha}/(1+\alpha^2))$. Inserting these expressions gives the equation for the rotation/translation part of the pressure (1, 2)

$$\frac{1}{12\mu}\partial_i(h^3\partial_i p_m) + V\alpha/2 = (U + x\dot{\alpha}/(1+\alpha^2)) \quad [8]$$

As a boundary condition, pressure is assumed to vanish anywhere outside the surface of the robot, as there is no surface here to support stresses.

For the electrokinetic contribution to the pressure, all the fluid velocities at interfaces are proportional to the electrokinetic slip. Thus $u_i^+ = \beta^+ E_i$ and $u_i^- = \beta^- E_i$. The resulting Reynolds equation for this part of the flow is:

$$\frac{1}{12\mu}\partial_i(h^3\partial_i p_{ek}) = \partial_i(h(\beta^+ + \beta^-)E_i)/2 + \beta^+ E_z(z=h) - \beta^+\alpha E_x(z=h) \quad [9]$$

We find all the terms on the right hand side cancel identically. First, we note that the last two terms on the right hand side cancel, since the no-flux condition on the electric field through the robot's body implies $\hat{n}\cdot\vec{E} \propto E_x\alpha - E_z = 0$. Likewise, the first term on the right hand side is zero via equation 1. Without source terms, the boundary condition of vanishing pressure outside the robot's body means the electrokinetic portion of the flow does not contribute to pressure and it sole forcing is through shear stress.

Since we are not interested in the pressure itself, but rather its integral over the robot's surface, we can numerically compute the forces and torques in equation 4 using the variational approach in (2, 3). Specifically, equation 8 implies the pressure also minimizes the functional

$$\langle[\frac{\partial_i p h^3 \partial_i p}{12\mu} - p(V\alpha/2 - U - \dot{\alpha}x/(1+\alpha^2))]\rangle \quad [10]$$

Thus, we approximate $p \approx (y^2 - (w/2)^2)\sum_{n=1}^{N}\sin[n\pi(x+l/2)/l]a_n$ where a_n are free parameters choosen to minimize equation 10. This expansion takes the first two terms of a Taylor series in the y coordinate, accounting for symmetry and boundary conditions, and expands pressure variation along the x-axis in terms of trigonometric functions that vanish at the ends of the robot. Inserting this form into equation 10 and minimizing reduces finding the pressure to a sparse linear algebra problem, which is efficient to solve numerically.

For the external forcing in equation 4, we assume $f_z = mg$ and introduce two phenomenological parameters $\tau = a\Delta\phi$ and $f_x = b\Delta\phi$. These parameters are motivated by additional experiments in which robots on their sides were observed to translate and rotate in proportion to the incident optical power.

To find the robot's equilibrium state, we insert the pressure expansion and external force parameters a into equation 4 with the optimal a_n. The three equations can be used to solve for the velocity in terms of the field and forcing parameters alone. This result can be reinserted into equation 4 to give two closed equations for the gap height and angle of attack evolution. We solve these two equations numerically (using Scipy's ODEInt Package) until they reach equilibrium to find the steady state speed, angle of attack, and velocity.

To produce the fits in Figure 2b and c, we used the Nelder-Meade simplex algorithm to simultaneously minimize the mean square error between the observed data and numerical solutions. Data and the model were written in dimensionless form, scaling all lengths by the robot's longest dimension l, all velocities by the viscous settling time $\mu l/mg$, and all forces by mg. The resulting system of equations has three free parameters: the scaling between velocity and electric field and the phenomenological torque and shear parameters a and b, which the solver adjusts to minimize error. All of the code used for solving for the pressure, integrating the ODE and fitting the data is included in the supplementary material.

D. Model for predicting κ. In an ideal differential drive system lateral motion is prohibited and the path of the robot can be described by its progression along a tangent circle with curvature κ. The robot travels with a body velocity V_b and the motor separation is given by 2δ. We write the following equations for the left and right motor velocities:

$$V_L = V_b(1+\delta\kappa) \quad [11]$$

$$V_R = V_b(1-\delta\kappa) \quad [12]$$

The body velocity V_b can be written as $\frac{V_L}{2} + \frac{V_R}{2}$. We solve for the curvature κ by subtracting V_L and V_R using this substitution for V_b.

$$\kappa = \frac{V_L - V_R}{(V_L + V_R)\delta} \quad [13]$$

We previously defined $\eta = (V_L - V_R)/(V_L + V_R)$, so we write κ as:

$$\kappa = \frac{\eta}{\delta} \quad [14]$$

While Equation 14 describes an ideal differential drive system, we find empirically that the direct relationship between light intensity and motor velocity requires an additional term, α, to capture the effect of optical power incident on one motor spilling over to the other motor due to imperfect hologram resolution. We extract this term experimentally by measuring the speed of

a motor when the optical power is incident on the motor and again when it the laser spot is offset by a distance δ. The ratio of these two values gives the adjustment factor for η. Thus, our equation of curvature accounting for optical effects is given by:

$$\kappa = \frac{\eta * \alpha}{\delta} \qquad [15]$$

Experimentally we find a value of $\alpha = 0.39$ for the devices shown in Figure 3 and Figure 4. To find the predicted values of the plot in Figure 3c, we calculate curvature κ for $\eta = [-1, 1]$, the entire range of possible values.

E. Control laws. We implement two controllers in Figure 3d and Figure 3e. In both demonstrations, the computer calculates a misalignment angle θ after every frame capture as the angle between the robot's heading and a vector that points from the robot's center of mass to the target position. In the first control scheme, if θ is increasing or larger than a user-defined threshold (here $15°$), the controller increases the power on the motor farther from the waypoint to realign the robot. If θ is decreasing, the power proportion is not changed. Using this rule, the controller maintains a misalignment angle of less than $15°$ over a majority of the path trace (Fig. 3d). With the same definition of θ, the second controller is a proportional controller that directs the robot by powering the left motor, the right motor, or both when θ is smaller than a user-defined threshold (here $7.5°$) for two consecutive frames. Additionally, this controller uses θ to weight the motor power. As seen in Figure 3e, sharp increases in θ after receiving a new waypoint results in the controller sending all available power to a single motor for turning.

F. Scaling up to more robots. A common goal of various microswimmer platforms is to build systems consisting of multiple devices that are capable of exhibiting swarm-like behaviors such as coordinated locomotion. In this work, we show that tying the propulsion system to onboard electronics turns robots into discrete devices that can easily operate separately from one another provided the electrical currents can be independently controlled. While here we do this directly using optics, integrating onboard circuits to regulate the motor currents and perform computation is a direct route towards building large swarms of independently controlled robots, each with its own onboard brain, controller, and propulsion system. To interact with robots, circuits for optical communication can enable the optical signal to be repurposed as a channel to interface with the robots individually. Using light, we can beam instructions to any number of devices in unison to send information, reprogram devices, or transfer data. Notably, when using the optical system in this way, the computation requirements do not significantly change when the system size increases as the runtime of the SLM system is dominated by two, scale-independent steps: Extraction of the phase map with the GS algorithm (4) and updating the SLM display with a new optical pattern (which is set by the switching speed of the SLM). In other words, we can send data or individual instructions to each robot in a swarm simultaneously by multiplexing light without worrying about computation time as the system size scales up.

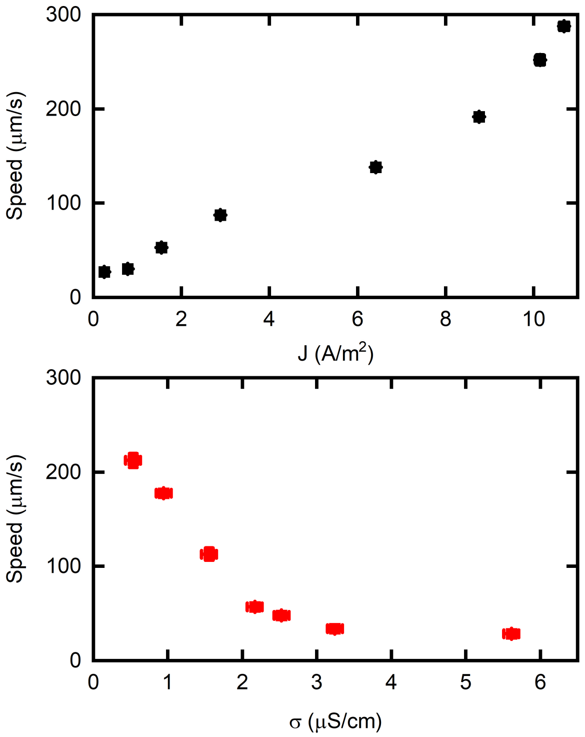

Fig. S1. Speed vs. current density and conductivity. Top: Robot speed as a function of current density for a fixed solution conductivity of 300 nS/cm in 5 mM hydrogen peroxide. Bottom: Robot speed as a function of conductivity in 5 mM hydrogen peroxide. Here current density also varies due to the changes in conductivity.

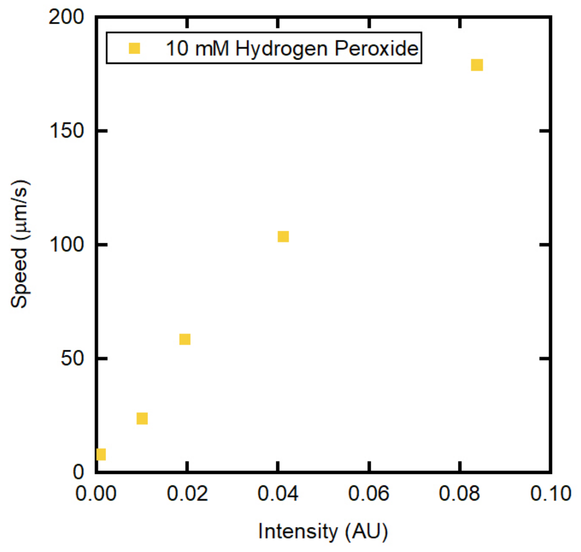

Fig. S2. Speed vs. intensity. Robot speed as a function of light intensity, i.e. optical power. For speeds below approximately 200 μm/s we find a linear relationship.

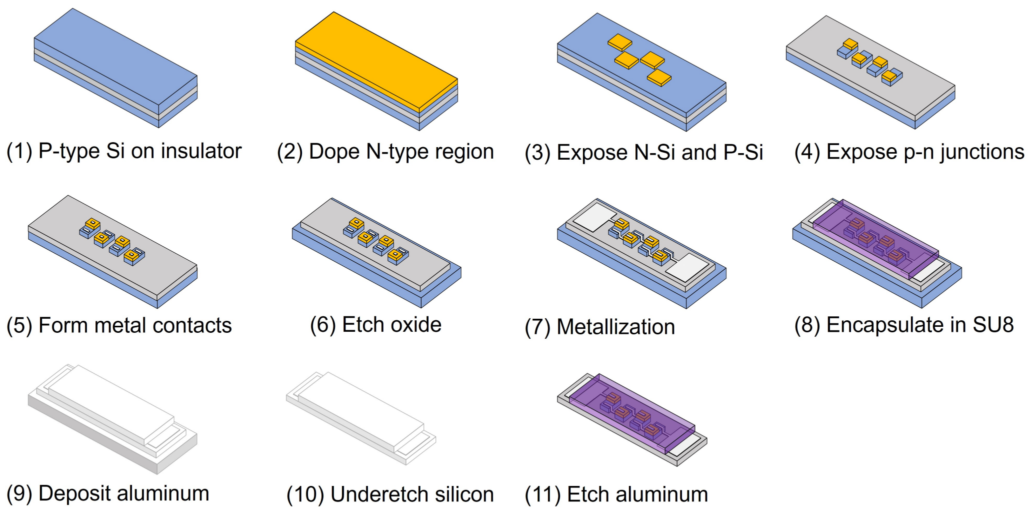

Fig. S3. Fabrication of a single motor. Steps (1)-(4) form discrete PVs through a series of doping and etching steps, Steps (5)-(7) deposit the electrical interconnects and actuator electrodes, and Steps (8)-(11) encapsulate and release the motors.

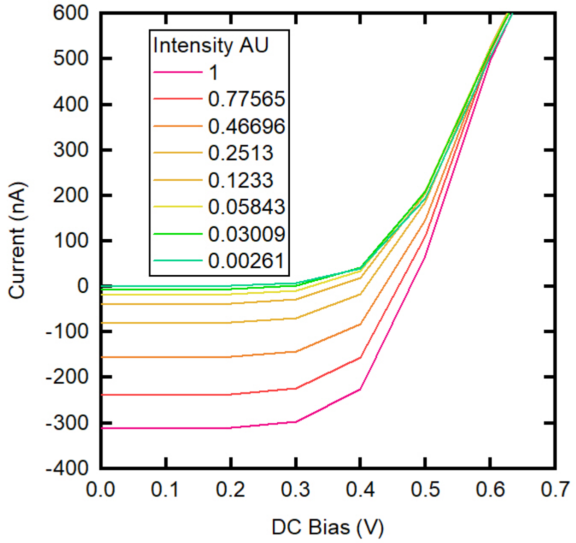

Fig. S4. PV I-V sweeps. Current vs voltage sweeps of a single PV under various illumination intensities, normalized to the maximum intensity of our light source.

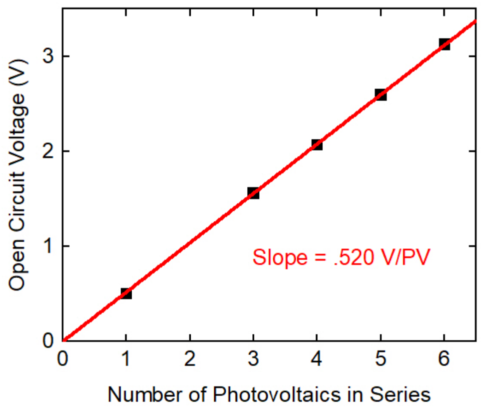

Fig. S5. Open circuit voltage. Open circuit voltage at maximum intensity as a function of the number of PVs wired in series.

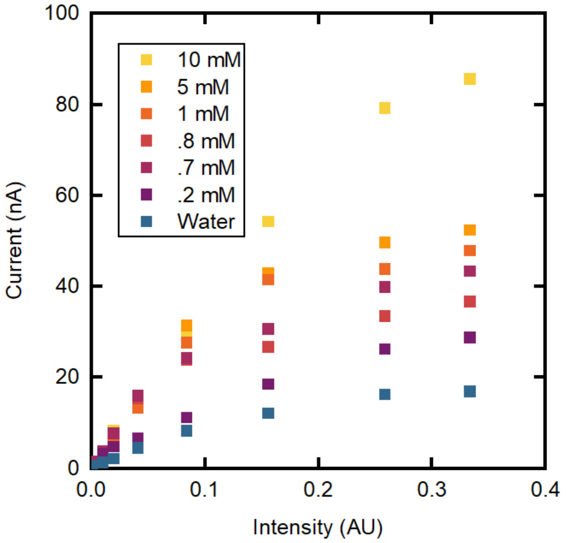

Fig. S6. PV output in different chemical environments. Current driven through various solutions by the PVs as a function of light intensity.

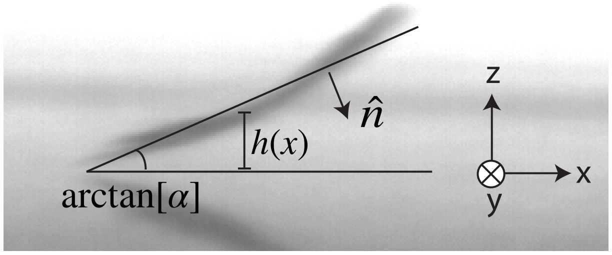

Fig. S7. Frame of reference for modeling propulsion. A schematic of the coordinates and approximation for geometry under the robot's body used in modeling the electric field and fluid flows.

Movie S1. A single motor under microscope illumination. (This video is in real time).

Movie S2. A single motor robot travels through an SU8 channel under microscope illumination. (This video is in real time)

Movie S3. A side-on view of a two motor robot under illumination. Due to asymmetry in the motors, the robot drives in a circle. At the end of the video, the driving illumination is turned off and the robot stops moving. (This video is in real time).

Movie S4. A controller autonomously pilots a robot to a waypoint by trimming the power on each engine. (This video has been sped up by 2x).

Movie S5. A controller autonomously pilots a robot through a series of waypoints to trace out a lemniscate. (This video has been sped up by 2x).

Movie S6. Given a list of target locations, robots autonomously travel to the nearest waypoint to form user-defined shapes. Shown here is a rectangle. (This video has been sped up by 2x).

Movie S7. Given a different list of target locations, robots rearrange to form other user-defined shapes such as triangles. (This video has been sped up by 2x).

Movie S8. Robots are assigned an individual list of waypoints to trace out separate paths simultaneously. (This video has been sped up by 2x).

Movie S9. Robots are assigned dynamic waypoints such as the location of another robot in the system and form a chain-like structure in which they follow their nearest neighbor. Additionally, the controller prevents robots from getting too close together, resulting in the stop-and-go behavior seen in this video. (This video has been sped up by 2x).

1. Software

The relevant code to solve for pressure, integrate the ODE, and fit the data is included on Github.

References

1. LG Leal, *Advanced transport phenomena: fluid mechanics and convective transport processes.* (Cambridge University Press, Cambridge), (2007) OCLC: 173817039.
2. AZ Szeri, *Fluid film lubrication.* (Cambridge University Press, Cambridge, U.K.), 2nd ed edition, (2011) OCLC: 719369575.
3. A Szeri, D Powers, Pivoted Plane Pad Bearings: A Variational Solution. *J. Lubr. Technol.* **92**, 466–472 (1970) _eprint: https://asmedigitalcollection.asme.org/tribology/article-pdf/92/3/466/5795764/466_1.pdf.
4. PJ Christopher, GSD Gordon, TD Wilkinson, Benchmarking the Gerchberg-Saxton Algorithm (2020) arXiv:2005.08623 [physics].
